# Numerical simulation of seasonality in the distribution and fate of pyrene in multimedia aquatic environments with Markov chains

**DOI:** 10.1038/s41598-017-10569-7

**Published:** 2017-08-29

**Authors:** Caiyun Sun, Liang Xu, Dazhi Sun, Libo Chen, Jiying Zou, Zhenxing Zhang

**Affiliations:** 1 School of Resources and Environmental Engineering, Jilin Institute of Chemical Technology, NO. 45 Chengde Street, Jilin, 132022 People’s Republic of China; 2 Institute of Grassland Science, Northeast Normal University, and Key Laboratory of Vegetation Ecology, Ministry of Education, Changchun Jilin, 130024 China

## Abstract

This case study investigated the distribution and fate of organic pollutants in aquatic environments based on laboratory experiments and modeling. Pyrene (Pyr) is a hydrocarbon pollutant with adverse effects on aquatic ecosystems and human health, and was thus selected for this case study. The movement of Pyr was primarily influenced by its sorption from water onto sediment, and its desorption from sediment into water. Its elimination was mainly via biodegradation by microorganisms in sediment and by volatilization from water into air. The transport and elimination rates for Pyr were considerably influenced by temperature and moisture. Results of modeling with Markov chains revealed that the elimination of Pyr from water/sediment systems was the most rapid under wet conditions. Under average conditions, a Pyr concentration of 100 μg/L of in water in such a system declined to a negligible level over 250 h. Under wet conditions, this decrease occurred over 120 h. Finally, under dry conditions, it took 550 h to achieve the same degree of elimination.

## Introduction

In China, the pollution of water and sediment with polycyclic aromatic hydrocarbons (PAHs) has grown into a significant issue^1^. Previous studies have indicated that PAHs in water and sediment present ecological and human health risks^2–5^. For pollution control and establishing toxicity risk assessment, it is essential to determine the distribution and fate of PAHs in aquatic environments (primarily water and sediment media) through data collection^6, 7^. Most studies to date have focused on the distribution and partitioning of PAHs in water/sediment systems based on field data^8, 9^.

Pyrene (Pyr) is a 4-ring PAH that is not subject to any environmental standards for water and sediment in China. This compound is widely distributed and persistent in the aquatic environment^10, 11^. From water and sediment, Pyr can enter the human body via the food chain, and may pose the risk of carcinogenicity^12^. In air/water/sediment systems, Pyr may be transported via sediment sorption and desorption, and may be eliminated from the system through the mechanisms of biodegradation, volatility, hydrolysis and photolysis^13–16^. The individual transport and elimination rates can be calculated based on models. However, for numerical simulation/modeling of Pyr distribution and its fate in air/water/sediment systems, a suitable and efficient environmental model must be selected to synergize with all of its transport and elimination mechanisms.

Markov chains, a well-established model, can describe a pollutant’s transport in multimedia environments with transition matrices^17^; the transport of each state is independent of the previous state and is stochastic^18^. The transport and elimination processes of Pyr in aquatic environments are independent of the previous state and are stochastic. For example, Sun *et al*. and Zhu *et al*. applied Markov chains to model the distribution and fate of phenanthrene and nitrobenzene in multimedia aquatic environments, with the results indicating that this assessment method was efficient and accurate^19, 20^. Accordingly, Markov chains were selected in this study to model the distribution and fate of Pyr in aquatic environments.

In this research, the Yinma River Basin (43°0′N–45°0′N, 124°30′E–126°0′E), which undergoes three distinctive moisture regimes, was selected as the study area. Because the basin witnesses seasonal variation in temperature and sunlight, the seasonality of the distribution and fate of Pyr should also be clarified. Thus, the main objectives of our research were to use laboratory experiments to investigate the transport and elimination pathways of Pyr in air/water/sediment systems and examine the seasonal variations in its transport and elimination rates. A secondary objective was to model the seasonal distribution and fate of Pyr in air/water/sediment systems with Markov chains.

## Results

### Simulation experiments

**Figure 1:**
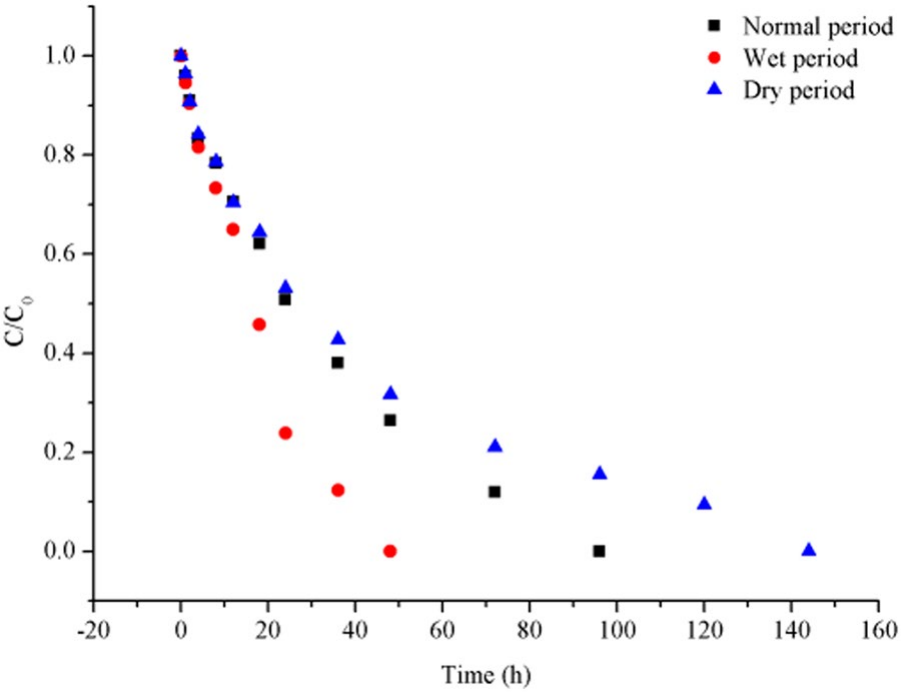
The initial and final relative concentrations of Pyr in water exposed to sorption processes in three moisture regimes.

All of the data in this study were based on laboratory experiments. Figure 1 illustrates the sorption processes of Pyr from water onto sediment in three moisture regimes, namely, wet, normal and dry. The highest sorption rate was observed in the wet period, and the lowest was observed in the dry period. As indicated in Table 1, under normal and dry conditions, sorption occurred at a relatively constant rate. In the dry period, the rate of sorption was highest in the first 48 h.

**Table 1:** Transport and elimination rates of Pyr in an air/water/sediment system.

	Normal period	Wet period	Dry period
Sorption (ng/mL/h)	0.0284	0.0578	<48 h 0.0231
			>48 h 0.0164
Desorption (ng/g/h)	<36 h 0.0538	<18 h 0.0775	<36 h 0.0278
	>36 h 0.0031	>18 h 0.0051	>36 h 0.002
	<18 h 0.0098	<12 h 0.021	<12 h 0.0076
Biodegradation (ng/g/h)	>18 h 0.0135	12–48 h 0.0373	12–72 h 0.0116
		>48 h 0.0227	>72 h 0.0061
Volatility (ng/mL/h)	0.0186	0.0542	0.0011

**Figure 2:**
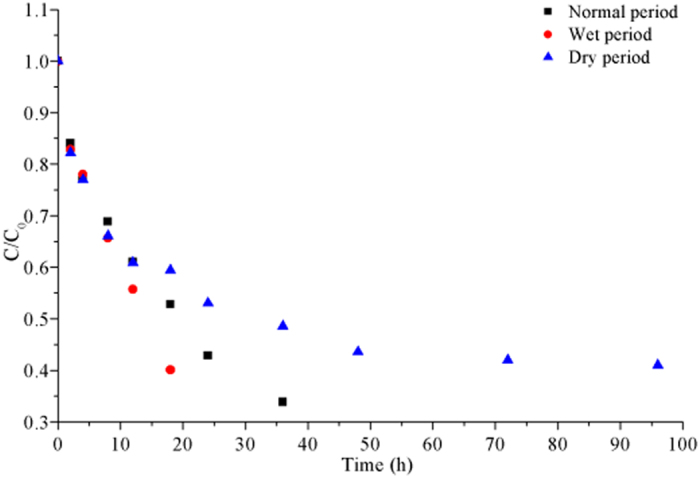
The initial and final relative concentrations of Pyr in water exposed to desorption processes in three moisture regimes.

Figure 2 illustrates desorption processes of Pyr from sediment into water in three moisture regimes. The desorption processes also varied across moisture regimes, and followed the same pattern as for sorption. Table 1 indicates that the desorption rates declined with time in all moisture regimes. During the wet period, the desorption rate declined rapidly after 18 h, while a similar decline took 36 h in the other two moisture regimes.

**Figure 3:**
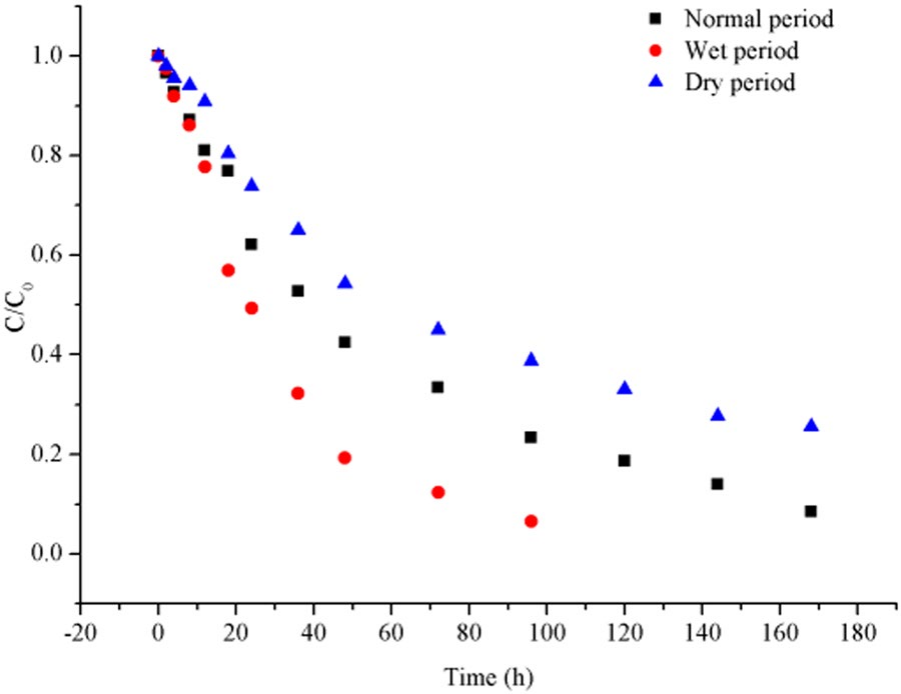
The initial and final relative concentrations of Pyr in sediment exposed to biodegradation processes in three moisture regimes.

Based on biodegradation experiments in three moisture regimes, the rate of biodegradation of Pyr by microorganisms in water was negligible. Therefore, this was not considered a significant pathway of elimination in water. Figure 3 displays the seasonal variation in biodegradation of Pyr by microorganisms in sediment. The biodegradation rate was correlated to moisture, which in turn was correlated to temperature. Table 1 indicates that in the normal period, the biodegradation process was slow at first but rapidly accelerated afterwards. The wet and dry periods exhibited a third stage whereby the rate of biodegradation decreased to very low levels and then stabilized.

**Figure 4:**
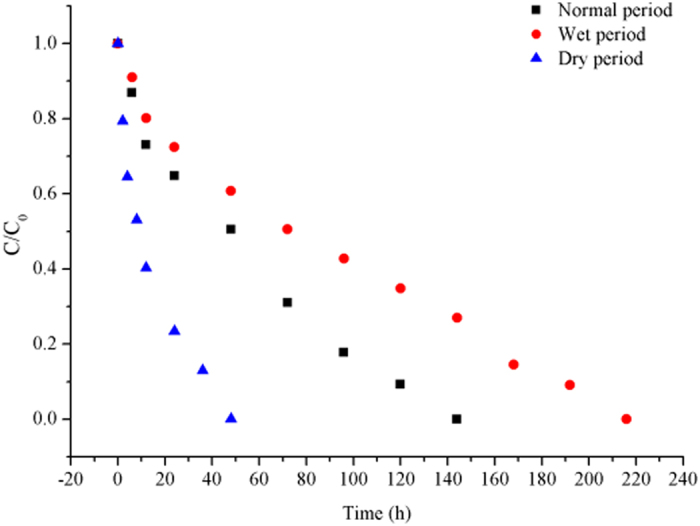
The initial and final relative concentrations of Pyr in water exposed to volatilization processes in three moisture regimes.

Laboratory experiments revealed that photolysis and hydrolysis rates were negligible across all moisture regimes. As shown in Fig. 4, volatility processes displayed seasonal variation. Table 1 indicates that the rate was the highest in the wet period, whereby 100 μg of Pyr per liter of water was volatilized into air in 48 h. In the dry period, it took 216 h for an equivalent amount to be volatilized.

### Numerical modeling of the seasonal distribution and fate of Pyr

**Figure 5:**
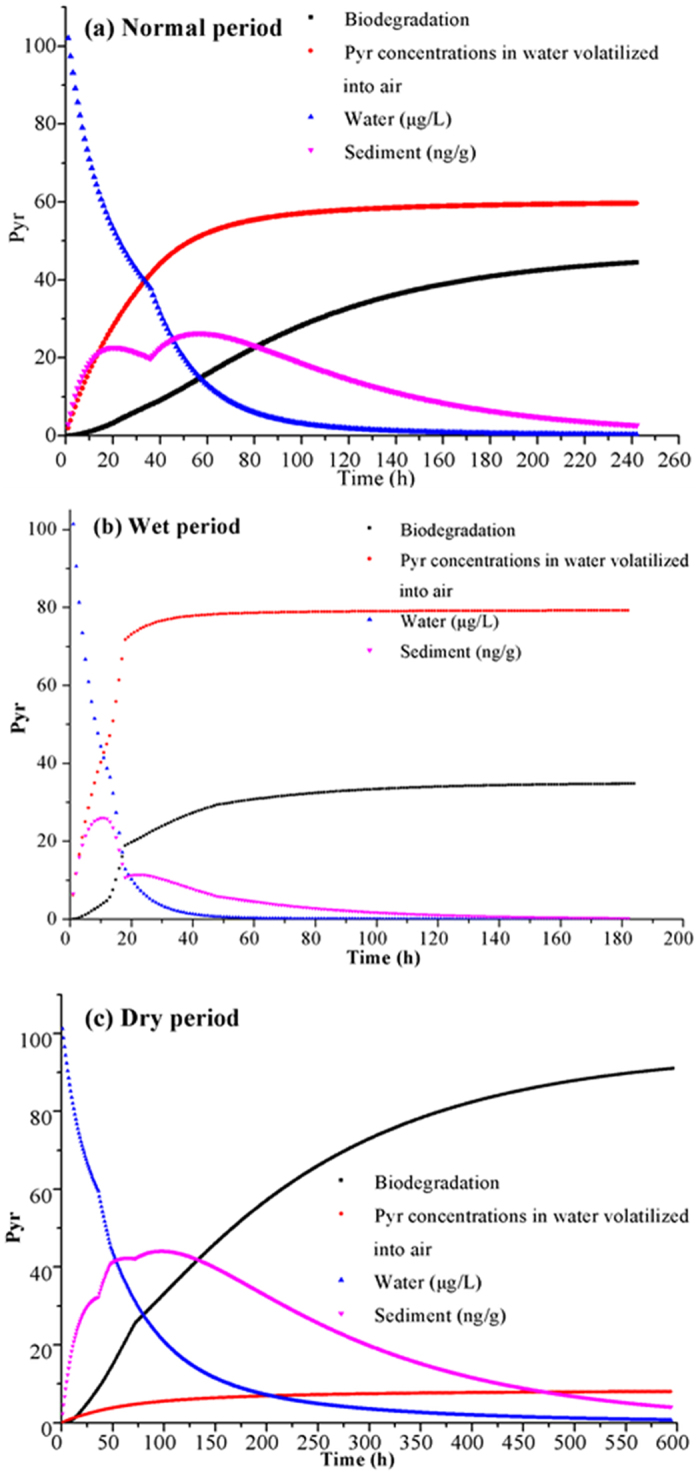
(**a**–**c**) Modeling of NP concentrations in four states with Markov chains in three moisture regimes.

Figure 5a–c represents modeled concentrations of Pyr in an air/water/sediment system over time, when exposed to biodegradation and volatilization processes with Markov chains. Changes in Pyr concentrations air, water and sediment were similar across all moisture regimes. In water, Pyr concentrations decreased rapidly at first, and then slowly thereafter. Simultaneously, Pyr concentrations in sediment increased rapidly at first, as a result of sorption. After equilibrium was attained, Pyr concentrations began to decrease slowly. Volatilization and biodegradation of Pyr from water and sediment, respectively, were rapid initially and later stabilized at steady rates.

In all of the three moisture regimes, the main elimination pathways of Pyr from a water/sediment system were volatility and biodegradation by microorganisms in sediment, but this exhibited seasonal variation. In the normal period, volatilization contributed to 57% of the Pyr eliminated, with the remaining 43% accounted for by biodegradation in sediment; in wet period. In contrast, volatilization accounted for 70% and 8% of Pyr elimination in the wet and dry periods, respectively.

Based on the results with application of Markov chains for Pyr in a water/sediment system, elimination rates displayed seasonal variation, with the highest rates in the wet period. In the normal period, water containing 100 μg/L of Pyr attained a negligible Pyr level after 150 h. The same degree of Pyr elimination from sediment took 250 h. In the wet period, the corresponding elimination times were 50 h and 120 h, respectively. Finally, in the dry period, the corresponding elimination times were 550 h and 600 h from water and sediment, respectively.

## Discussion

In this study, sorption, desorption, biodegradation by microorganisms and volatility were the key transport and elimination pathways for Pyr in air/water/sediment systems. Its transport and elimination rates were influenced by temperature. At higher temperatures, Pyr exhibits increasingly rapid molecular movements in all environmental media^21, 22^. Therefore, sorption, desorption and volatilization rates were all the highest in the warm, wet period, and the lowest in the cool, dry period. Because sediment provides sufficient sorption sites, the apparent sorption processes in the normal and wet periods occurred within one stage and at a relatively constant rate. In contrast, Pyr sorption processes in the dry period exhibited two stages. Initially, the sorption rate decreased considerably as the number of sorption sites decreased. Subsequently, the rate plateaued.

Since the number and activities of microorganisms are positively correlated to temperature^19^, the biodegradation rate was the highest in the wet period. The biodegradation processes also underwent more than one stage, because of a lag in microbial activity^20^. Therefore, the rate of biodegradation gradually increased as microbial populations in sediment increased. After reaching a peak, the biodegradation rate decreased subsequently.

Previous studies^23–27^ have indicated that some dynamic models are useful in revealing processes such as pollutant distribution and fate. However, Markov chains were selected as it can describe a pollutant’s transport in multimedia environments with transition matrices, where the transport of each states is independent of the previous state and is stochastic. These accurately describe the processes of Pyr transport and transformation. The transport and elimination processes represent seasonal variations, with the rate of both rates displaying positive correlations with temperature (Table 1). Therefore, according to the results for Markov chains, the elimination of Pyr from water/sediment systems revealed seasonal variation. The rate of Pyr elimination from a water/sediment system was highest in the wet period and lowest in the dry period. Changes in Pyr in all states were similar across moisture regimes (Fig. 5a–c). Once Pyr entered the air/water/sediment system, a part of the Pyr in water quickly adsorbed onto sediment and a part volatilized into air, resulting in a quick decrease in Pyr concentrations in water and a corresponding rapid increase in Pyr concentration in sediment. After some time, sorption reached an equilibrium as evidenced by a decrease in the sorption rate. Subsequently, Pyr adsorbed on sediment began desorbing into water, resulting in a decrease in sediment Pyr concentrations and a near-plateau in water Pyr concentrations. The elimination pathways for Pyr from water/sediment systems were mainly through biodegradation by microorganisms in sediment and volatilization from water. However, variation in the biodegradation and volatilization rates across moisture regimes resulted in different proportions of Pyr being lost via biodegradation or volatilization, depending on the moisture regime.

Investigating the distribution and fate of organic pollutants in multimedia environments is essential for toxicity risk assessment, and pollution control and remediation, but the majority of studies on these aspects have been based on field data and the physicochemical properties of pollutants. In this study, Markov chains analysis was employed for numerical modeling of Pyr distribution and fate in air/water/sediment systems.

## Methods

### Laboratory experiments

The Yinma River Basin (43°0′N–45°0′N, 124°30′E–126°0′E), located in Jilin Province, China, was selected as the reference area. Water and sediment samples were collected from this basin. The initial concentrations of Pyr in these samples were negligible. In the Yinma River Basin, the average water temperatures are 13 °C during the average period (spring and autumn), 25 °C in the wet period (summer) and 5 °C in the dry period (winter). All the laboratory experiments were designed to mimic these conditions.

Water samples were filtered through 0.45-μm and 3-μm (for biodegradation experiments) glass fiber filters, while sediment samples were ground and passed through 100-cm sieves. All water and sediment samples were stored at −4 °C. With the exception of samples used in the biodegradation experiments, all samples and equipment were sterilized.

#### Sorption and desorption experiments

50 g of sediment was placed in a 250-ml brown conical flask filled with a water sample spiked with 100 μg/L of Pyr. A negligible headspace was maintained to minimize volatilization and the flask was capped with 8 layers of sterile gauze. The flak was horizontally shaken on an orbital shaker at 150 rpm, after which 1 mL of the supernatant was sample at specific time intervals for high performance liquid chromatography (HPLC) analysis within 24 h.

For desorption experiments, sediment containing 100 ng/g of Pyr was prepared by spiking sediment with Pyr solution. Next, 50 g of this sediment sample was mixed with 200 mL of sampled water in a flask. The sediment/water mixture was horizontally shaken on an orbital shaker at 150 rpm. At specific time intervals, 1 mL of the supernatant was sampled for HPLC analysis within 24 h. Every 12 h, supernatant was completely removed via triple centrifugation (2800 rpm for 20 min) and 200 mL of water was added again. This process continued until the Pyr concentration in the water decreased to below the detection limit.

#### Biodegradation by microorganisms in sediment

Sterilized and unsterilized sediment samples containing 100 ng/g Pyr were prepared. For both types of samples, 200 g was placed in brown Teflon-lined vials. The vials were tightly capped with 8 layers of sterile gauze to create an anaerobic atmosphere. At predetermined time intervals, 2 g of sample was sampled from each of the vials for HPLC analysis within 24 h.

#### Volatility, photolysis, hydrolysis and biodegradation in water

Preliminary experiments indicated that photolysis, hydrolysis and biodegradation by indigenous photoheterotrophic microorganisms and microorganisms in water were not significant elimination pathways for Pyr from water/sediment systems. The methods for these experiments are referenced in SI 3.4.

#### Volatility experiments

Pyr solution (100 µg/L), prepared from a water sample, was placed in a 2 L brown breaker exposed to air. The solution was stirred at 150 rpm with magnetic stirrers in the dark (to avoid loss by photolysis). At predetermined intervals, 1 mL of solution was sampled for HPLC analysis.

### Quality controls

All data were subjected to rigorous quality controls. Standard solutions of Pyr were used to validate methods and produce calibration curves with regression coefficients higher than 0.996. All laboratory experiments were conducted in duplicate, with all relative standard deviations being less than 15%. Five parallel experiments were conducted to assess the efficiencies of Pyr recovery from sediment by spiking samples with Pyr standard solutions. The Pyr recovery rates were 80–108%, indicating that the extraction method for Pyr from sediment was efficient.

#### Extraction and HPLC quantification methods

The extraction and HPLC quantification methods for Pyr in sediment were as specified in SI 3.6 and 3.7, respectively.

### Markov chains

Markov chains represent a well-established model for a stochastic process. This theory, uses transition matrices to describe the transfer of a pollutant in multimedia environments through one or more steps. Pollutant transferring from one environmental medium to another is considered as being in a transient state, whereas a pollutant disappearing from a multimedia environment is regarded as being in an absorption state. The likelihood of a pollutant transferring from an absorption states to a transient states is zero^13, 19, 20^.

Assuming that Markov chains exist in *n* finite states, the state space can be expressed as U = (U_1_ + U_2_ + U_3_ + ...+ U_n_). P_ij_ (t) represents the transition matrix to describe the possibilities of the transition of state *i* to state *j* over a certain time interval (t_0_). The transition matrix constituted by P*ij* (*i*, *j* = 1, 2, 3 ...) can be expressed as follows:

1 $${\rm{p}}=(\begin{array}{cccc}{p}_{11} & {p}_{12} & \cdots  & {p}_{1n}\\ {p}_{21} & {p}_{22} & \cdots  & {p}_{2n}\\ \vdots  & \vdots  & \ddots  & \vdots \\ {p}_{n1} & {p}_{n2} & \cdots  & {p}_{nn}\end{array})$$

If the pollutant has *k* transient states and *r* absorption states, the transition matrix can be described as follows:

2 $${\rm{p}}=(\begin{array}{cc}{{\rm{I}}}_{{\rm{r}}\times {\rm{r}}} & {{\rm{O}}}_{{\rm{r}}\times {\rm{k}}}\\ {{\rm{R}}}_{{\rm{k}}\times {\rm{r}}} & {{\rm{Q}}}_{{\rm{k}}\times {\rm{k}}}\end{array})$$

where I_r×r_ represents the transition possibilities of transferring from absorbing states to absorbing states; O_r×k_ represents the transition possibilities of transferring from absorbing states to transient states; R_k×r_ represents the possibilities of the pollutant disappearing and transferring over each hour; Q_k×k_ is represented as the possibilities of the pollutant transferring from one environmental medium to another over each hour.

If the pollutant transfers from one state to another over *t* + *t*_*0*_, the concentrations of the pollutant in and transferring to any states can be calculated by equation (3):

3 $${{\rm{U}}}_{({\rm{t}}+{\rm{t}}0)}={{\rm{U}}}_{({\rm{t}})}\times {\rm{P}}$$

In this study, sorption and desorption were transient states for Pyr, while biodegradation by microorganisms in sediment and volatilization from water were adsorption states. Accordingly, volatilization, biodegradation, solvation and sorption were designated as states 1, 2, 3 and 4, respectively. Based on transport and elimination rates (Table 1), the transient matrices were expressed as SI 3.8. The matrices for the Pyr distributions in four states were respectively expressed as: U (0) = (0 0 102.05 0) for the normal period; U (0) = (0 0 101.38 0) for the wet period; and U (0) = (0 0 101.24 0) for the dry period. The concentrations of Pyr every hour were calculated with equation (1).

## Electronic supplementary material

Supplementary file
