# Supplementary material for: Numerical simulation of seasonality in the distribution and fate of pyrene in multimedia aquatic environments with Markov chains
Source: Sci Rep. 2017 Aug 29;7:9796. doi: 10.1038/s41598-017-10569-7 (PMC5575142; doi:10.1038/s41598-017-10569-7)
Supplement: Supplementary file 1 — Supplementary file [file 41598_2017_10569_MOESM1_ESM.pdf]

Numerical simulation of seasonality in the distribution and fate of pyrene in multimedia aquatic environments with Markov chains

Caiyun Sun[a] Liang Xu[a]* Dazhi Sun[a]* Libo Chen[a] Jiying Zou[a] Zhenxing Zhang[b]

Affiliation: [a]School of Resources and Environmental Engineering, Jilin Institute of Chemical Technology, NO. 45 Chengde Street, Jilin 132022, People's Republic of China

[b] Institute of Grassland Science, Northeast Normal University, and Key Laboratory of Vegetation Ecology, Ministry of Education, Changchun, Jilin 130024 China

Mrs. Caiyun Sun: Email: 437621315@qq.com

Mr. Liang Xu: Email: xlsdydnl@126.com

Pro. Dazhi Sun: Email: sundazhi@jlict.edu.cn

Pro. Libo Chen: Email: 2031259758@qq.com

Mrs. Jiying Zou: Email: 670186912@qq.com

Dr. Zhenxing Zhang: Email: zhangzx725@nenu.edu.cn

***Corresponding authors:** Liang Xu and Dazhi Sun

3.4 Photolysis, hydrolysis and biodegradation experiments

Photolysis: 100 μg/L of Pyr solution dissolving in natural water was filled with a quartz petri dish, and the dish was capped with a quartz lid tightly to avoid volatility loss. The experiments were conducted on the roof of the building in the region of Yinma River Basin. At determined time intervals, 0.5 mL of solution was taken for HPLC analysis with 24 h.

Hydrolysis: 100 μg/L of solution dissolving in natural water was filled with a 100-mL Teflon-capped brown vial and capped tightly with 8 layers of sterile gauzes. The solution was stirred at 150 rpm with a magnetic stirrer, and 0.5 mL of solution was taken for HPLC analysis within 24 h at determined time intervals.

Biodegradation by microorganisms in water: Two-100 μg/L of solutions dissolving in sterilized and unsterilized natural water were respectively added in two 250-mL Teflon-capped brown glass vials. The vials were capped tightly with 8 layers of sterile gauzes to avoid the volatility loss, and the solutions were stirred at 150 rpm with a magnetic stirrer. 0.5 mL of solution was taken for HPLC analysis at determined time intervals.

To clarify whether Pyr was biodegraded by indigenous photoheterotrophic microorganisms in natural water, experiments for biodegradation of Pyr by indigenous photoheterotrophic microorganisms in natural water were conducted. Two-100 μg/L of solutions dissolving in unsterilized and sterilized natural water were filled with quartz petri dishes respectively, and the dishes were capped with quartz lids to avoid volatility loss. The experiments were conducted on the roof of the building in the region of Yinma River Basin. 0.5 mL of solution was taken for HPLC analysis within 24 h at determined time intervals.

All the simulation experiments and control experiments were duplicated, and conducted at the same time.

3.6 Extraction method for Pry in sediment

The extraction method of Pyr in sediment was followed as a published article[1]. 2 g of sediment were thoroughly mixed with 2 g of anhydrous sodium sulfate, and were added with 30 mL of mixture of n-hexane/dichloromethane (1:1/v:v), then were extracted in ultrasonic bath for 30 min for three times, and the extracts were reduced to 1 mL by a rotary vacuum evaporation apparatus. 1 mL of extract and 10 mL of n-hexane were successively passed through a silica/anhydrous sodium sulphate column, and elutes were discharged. After that, 30 mL of mixture of n-hexane/dichloromethane (1:1/v:v) were passed through the column, the elutes containing Pyr were collected, and reduced to dryness, then dissolved with 1 mL of methanol for HPLC analysis. In the whole extraction process, the flow rates were controlled at 6 mL/min under vacuum.

Recovery rates were done before conducting extraction, and were within 80-108%. The results for recovery rates indicated that the extraction method for Pyr in sediment was efficient.

3.7 HPLC quantification method

The Pyr concentrations in the sediment and water were quantified with a HPLC system equipped with a symmetry reversed-phase C18 column (4.6 × 150 mm). Pyr was detected with a fluorescence detector with an excitation wavelength of 270 nm and an emission wavelength of 390 nm. The mobile phase (isocratic) was methanol/water (80/20, v/v), and flow rate was 1 mL/min. The detection limits were below 2 μg/L for water and below 2 ng/g for sediment.

3.8 Transient matrices

Normal period: 0 - 18 h $P = \left(\begin{array}{c|ccc} 1 & 0 & 0 & 0 \\ 0 & 1 & 0 & 0 \\ 0 & 0.0186 & 0.9462 & 0.0284 \\ 0.0098 & 0 & 0.0538 & 0.9716 \end{array}\right)$

18 - 36 h $P = \left(\begin{array}{c|ccc} 1 & 0 & 0 & 0 \\ 0 & 1 & 0 & 0 \\ 0 & 0.0186 & 0.9462 & 0.0284 \\ 0.0135 & 0 & 0.0538 & 0.9716 \end{array}\right)$

After 36 h $$P = \left(\begin{array}{c|ccc} 1 & 0 & 0 & 0 \\ 0 & 1 & 0 & 0 \\ 0 & 0.0186 & 0.9969 & 0.0284 \\ 0.0135 & 0 & 0.0031 & 0.9716 \end{array}\right)$$

Wet period: 0 - 12 h $$P = \left(\begin{array}{c|ccc} 1 & 0 & 0 & 0 \\ 0 & 1 & 0 & 0 \\ 0 & 0.0542 & 0.9225 & 0.0578 \\ 0.021 & 0 & 0.0775 & 0.9422 \end{array}\right)$$

12 - 18 h $$P = \left(\begin{array}{c|ccc} 1 & 0 & 0 & 0 \\ 0 & 1 & 0 & 0 \\ 0 & 0.0542 & 0.9225 & 0.0578 \\ 0.0373 & 0 & 0.0775 & 0.9422 \end{array}\right)$$

18 - 48 h $$P = \left(\begin{array}{c|ccc} 1 & 0 & 0 & 0 \\ 0 & 1 & 0 & 0 \\ 0 & 0.0542 & 0.9949 & 0.0578 \\ 0.0373 & 0 & 0.0051 & 0.9422 \end{array}\right)$$

After 48 h $$P = \left(\begin{array}{c|ccc} 1 & 0 & 0 & 0 \\ 0 & 1 & 0 & 0 \\ 0 & 0.0542 & 0.9949 & 0.0578 \\ 0.0227 & 0 & 0.0051 & 0.9422 \end{array}\right)$$

Dry period: 0 - 12 h $$P = \left(\begin{array}{c|ccc} 1 & 0 & 0 & 0 \\ 0 & 1 & 0 & 0 \\ 0 & 0.0011 & 0.9722 & 0.0231 \\ 0.0076 & 0 & 0.0278 & 0.9769 \end{array}\right)$$

12 - 36 h $$P = \left(\begin{array}{c|ccc} 1 & 0 & 0 & 0 \\ 0 & 1 & 0 & 0 \\ 0 & 0.0011 & 0.9722 & 0.0231 \\ 0.0116 & 0 & 0.0278 & 0.9769 \end{array}\right)$$

36 - 48 h $$P = \left(\begin{array}{c|ccc} 1 & 0 & 0 & 0 \\ 0 & 1 & 0 & 0 \\ 0 & 0.0011 & 0.998 & 0.0231 \\ 0.0116 & 0 & 0.002 & 0.9769 \end{array}\right)$$

48 - 72 h $$P = \left(\begin{array}{c|ccc} 1 & 0 & 0 & 0 \\ 0 & 1 & 0 & 0 \\ 0 & 0.0011 & 0.998 & 0.0164 \\ 0.0116 & 0 & 0.002 & 0.9836 \end{array}\right)$$

After 72 h $$P = \left(\begin{array}{c|ccc} 1 & 0 & 0 & 0 \\ 0 & 1 & 0 & 0 \\ 0 & 0.0011 & 0.998 & 0.0164 \\ 0.0061 & 0 & 0.002 & 0.9836 \end{array}\right)$$

References

1. Sun C., Zhang J., Ma Q., Zhang F,, Chen Y. Risk assessment of polycyclic aromatic hydrocarbons (PAHs) in sediments from a mixed use reservoir. Hum Ecol Risk Assess. 22(2):448-453 (2016).
